# Comparative Analysis of Machine Learning Methods to Predict Growth of *F. sporotrichioides* and Production of T-2 and HT-2 Toxins in Treatments with Ethylene-Vinyl Alcohol Films Containing Pure Components of Essential Oils

**DOI:** 10.3390/toxins13080545

**Published:** 2021-08-05

**Authors:** Eva María Mateo, José Vicente Gómez, Andrea Tarazona, María Ángeles García-Esparza, Fernando Mateo

**Affiliations:** 1 Department of Microbiology, School of Medicine, 46010 Valencia, Spain; Eva.mateo@uv.es; 2 Department of Microbiology and Ecology, University of Valencia, 46100 Burjassot, Spain; J.vicente.gomez@uv.es (J.V.G.); andrea.tarazona@uv.es (A.T.); 3 Department of Pharmacy, School of Health Sciences, University Cardenal Herrera CEU, 03204 Elche, Spain; maria.garcia2@uchceu.es; 4 Department of Electronic Engineering, ETSE, University of Valencia, 46100 Burjassot, Spain

**Keywords:** *Fusarium sporotrichioides*, ethylene-vinyl alcohol copolymers, essential oil pure components, machine learning, fungal growth, T-2 toxin, HT-2 toxin, predictive microbiology

## Abstract

The efficacy of ethylene-vinyl alcohol copolymer films (EVOH) incorporating the essential oil components cinnamaldehyde (CINHO), citral (CIT), isoeugenol (IEG), or linalool (LIN) to control growth rate (GR) and production of T-2 and HT-2 toxins by *Fusarium sporotrichioides* cultured on oat grains under different temperature (28, 20, and 15 °C) and water activity (a_w_) (0.99 and 0.96) regimes was assayed. GR in controls/treatments usually increased with increasing temperature, regardless of a_w_, but no significant differences concerning a_w_ were found. Toxin production decreased with increasing temperature. The effectiveness of films to control fungal GR and toxin production was as follows: EVOH-CIT > EVOH-CINHO > EVOH-IEG > EVOH-LIN. With few exceptions, effective doses of EVOH-CIT, EVOH-CINHO, and EVOH-IEG films to reduce/inhibit GR by 50%, 90%, and 100% (ED_50_, ED_90_, and ED_100_) ranged from 515 to 3330 µg/culture in Petri dish (25 g oat grains) depending on film type, a_w_, and temperature. ED_90_ and ED_100_ of EVOH-LIN were >3330 µg/fungal culture. The potential of several machine learning (ML) methods to predict *F. sporotrichioides* GR and T-2 and HT-2 toxin production under the assayed conditions was comparatively analyzed. XGBoost and random forest attained the best performance, support vector machine and neural network ranked third or fourth depending on the output, while multiple linear regression proved to be the worst.

## 1. Introduction

*Fusarium* is one of the fungal genera with the greatest impact in food mycology. Infection with *Fusarium* spp. commonly results in a reduction of the quality and yield of the crop. Additionally, many of these species are capable of producing toxic secondary metabolites with adverse effects on human and animal health. *Fusarium sporotrichioides* and *F. langsethiae* belong to the indigenous *Fusarium* community of cereal grains [1]. These two are closely related species and have similar secondary metabolite spectra [2,3]. Both have been reported to produce important levels of type-A trichothecenes, such as T-2 and HT-2 toxins (T-2 and HT-2), among others [2,4,5,6,7]. The toxicity of trichothecenes is mainly due to their ability to inhibit protein synthesis and, at higher doses, they can also inhibit DNA and RNA synthesis acting, predominantly, on actively dividing tissues such as bone marrow, lymph nodes, thymus and intestinal mucosa. T-2 also shows unspecific systemic effects, such as body weight reduction, and induces liver damage, reproductive toxicity, neurotoxicity, as well as haematotoxic and immunotoxic effects [8]. T-2 is rapidly metabolized to a large number of products, HT-2 being a major metabolite [9,10]. It has been reported that acute toxicity of T-2 and HT-2 in vivo are within the same range [8]. Thus, indicative levels for the sum of T-2 and HT-2 in cereals and cereal products have been established [11].

Several authors have documented the occurrence of T-2 and HT-2 in diverse cereals and cereal products, including conventionally and organically produced oats, barley, wheat or maize. In general, oats is the cereal with the highest levels of both toxins, especially, in countries of central and northern Europe [12,13,14,15,16,17,18]. The causative agent of this health risk in these countries is *F. langsethiae*. The physicochemical conditions (mainly water activity (a_w_)/relative humidity and temperature), including antifungal treatments, that affect the growth of *F. langsethiae* and T-2 and HT-2 production have been reported [3,19,20]. However, in the case of *F. sporotrichioides*, little is known about its physicochemical requirements [3,5,21,22], which could explain its geographical distribution, a complex process currently not fully understood.

In recent years, as food safety has received increasing attention, the demand for “natural” antimicrobial agents has increased and essential oils (EOs) have been the subject of numerous researches as an important source of eco-friendly food preservatives with great health benefits [23]. The biological activities (antibacterial, antifungal, antioxidant and anti-pest abilities) of plant EOs and their preservative potential in food systems has been verified [23,24,25,26], coming to the conclusion that they are potential alternatives to synthetic additives [27]. EOs have been given a “generally recognized as safe” (GRAS) status. Indeed, some flower EOs and their components have been approved by the United States Food and Drug Administration (FDA) and accepted by the European Commission (EC) as additives. They are, for example, clove EO, thyme EO, vanillin, carvone, eugenol, linalool, citral, cinnamaldehyde, or limonene [28], among others. In order to maintain their biological activity, reduce their volatility, increase their effective utilization rate and minimize the adverse effects of their odor, EOs need to be encapsulated in a suitable delivery system for food applications. Direct mixing into packaging substrates is the most commonly studied method [29,30,31]. Active packaging (AP) mainly refers to packaging technology that modifies the conditions of the packaged foods to upgrade its sensorial or safety characteristics and to extend its shelf life by protecting food from internal and external environmental factors while maintaining its quality. This improvement can be carried out by the inherent properties of the polymers used to manufacture the bags or pouches or by addition of active agents to the original packaging material or in the headspace of the AP [32]. Antimicrobial packaging is a type of AP that not only extends the shelf life of food but also helps maintain its quality [33].

Ethylene-vinyl alcohol copolymers (EVOH) are thermoplastic polymers with application in many industrial sectors including AP. EVOH properties are dependent on the copolymerization ratio of ethylene to vinyl alcohol. In particular, the copolymers with low ethylene contents (below 32 mol% ethylene) have outstanding barrier properties to O_2_ and aromas under dry conditions, compared to other polymers. In addition, EVOH copolymers exhibit a high transparency and interesting mechanical properties [34,35,36]. Recently, the ability of EVOH films supplemented with EOs or their pure components to control the growth of some toxigenic fungi and mycotoxin production has been reported [37,38,39]. Plastic films supplemented with EOs can be considered as antimicrobial packaging materials and also act as antioxidants. For example, EVOH containing garlic extract and bread aroma proved useful against *Penicillium expansum* and were used as coating material on polyethylene (PE) films to increase the shelf life of sliced bread [29,40].

Cereal grain is dried just after harvest to decrease the moisture content to a safe level which is considered to be equivalent to the equilibrium moisture of the grain at 75% relative humidity and 25 °C. This moisture content varies slightly for the different cereal grains, ranging between 13% and 15% [41]. It is crucial to preserve these commodities from fungal deterioration and to keep them free from contaminants such as mycotoxins by storing them in appropriate plastic bags, including airtight silo-bags [41,42], or hermetic containers.

Research on possible applications of bioactive films containing EOs or their components in the control of toxigenic fungi and mycotoxins in food is extraordinarily scarce and there are no previous studies on the effect of EVOH bioactive films to avoid or delay growth of *F. sporotrichioides* and production of T-2 and HT-2 in oat grains, other cereal grains, or their derivatives.

Machine learning (ML) is a branch of data analysis that enables computer programs to learn from data provided to the program and to identify patterns and associations that are too complex to be determined by the human brain [43]. ML is used to analyze large data sets and identify patterns in the data that happen to be associated with known or unknown outcomes. ML methods involve many disciplines, such as probability theory, statistics, approximation theory, etc. [44]. They can be mainly supervised or unsupervised. In supervised ML, the input data (or features) are labeled according to a known outcome in which the researcher is interested. In supervised ML, a human first categorizes each data in a training set that encompasses features and results (outcomes). The supervised ML goal is to find a model that best relates the input and output data. For example, an image of a Petri dish containing a culture medium could be considered as “growth present” or “growth absent”. A computer develops an algorithm to classify each image and compares the algorithm’s accuracy with the supervised “truth”; usually, initial results do not provide adequate performance and the algorithm will iteratively adjust its parameters by trial and error to optimize accuracy [45]. In this process, a validation set is used to develop the final model. Different methods can be used for the evaluation (simple holdout, k-fold cross-validation, leave-one-out cross-validation, etc.). In these methods, some data remain hidden during the training process. Two problems can arise during model development and should be avoided: underfitting and overfitting [46]. One of the best ways to avoid/minimize overfitting is the k-fold cross-validation method, in which the training dataset is split into k (usually 10) subsets. Then, a holdout training method is repeated k times, such that each time, one of the k subsets is used as the validation set and the remaining k-1 subsets form a training set. All observed values in the train subset are used for both training and validation, and each observation is used for validation exactly once. The parameter used for error estimation, for example, the root-mean square error (RMSE) is averaged over all 10 trials. This decreases bias and variance [39]. To assess the generalization of the model, a test set not used in the training task is used to evaluate the developed model. Supervised ML can be used in classification or prediction analysis. Common supervised learning algorithms are regression analysis (linear or logistic) and statistical classification [44]. Other common algorithms are support vector machines (SVM), artificial neural networks (NN), decision trees, k-nearest neighbors (kNN), random forest (RF), and gradient boosting algorithms, among which XGBoost (extreme gradient boosting) is highlighted. XGBoost is a decision-tree-based ensemble ML algorithm that utilizes a gradient boosting framework. Gradient boosting is a ML technique for regression and classification problems, which produces a prediction model in the form of an ensemble of weak prediction models, typically decision trees. It builds the model in a stage-wise fashion like other boosting methods, and it generalizes them by allowing optimization of an arbitrary differentiable loss function. The XGBoost implementation uses a regularized model formalization to control overfitting, which gives it better performance.

The usage of these methods in health sciences, like microbiology, has been increasing very fast in recent years. However, the application of ML in the field of predictive mycology, including prediction of mycotoxin production, is scarce. NN, both multi-layer perceptrons and radial-basis networks, have been applied to predict fungal growth in food [47,48] and have proven to be effective tools to predict ochratoxin A production by *Aspergillus carbonarius* and deoxynivalenol production by *F. culmorum* [49,50]. RF are ensembles of decision trees that can be used as nonlinear regression algorithms [51]. They have been applied in mycology for prediction [39,44,52,53]. XGBoost has also proven useful in previous mycological studies [39,53].

The aims of this multidisciplinary study were: (1) to assess the efficacy of EVOH films incorporating cinnamaldehyde (CINHO), citral (CIT), isoeugenol (IEG), or linalool (LIN) on the control of *F. sporotrichioides* growth and the production of T-2 and HT-2 in oat grains under different temperature/water activity (a_w_) regimes with the final objective of exploring its suitability as antimicrobial packaging material for storing oat grains and derivatives, and (2) to comparatively evaluate the potential of several ML methods to predict the growth rate (GR) of *F. sporotrichioides* and production of T-2 and HT-2 under the assayed conditions.

## 2. Results

### 2.1. Effect of EVOH Films Incorporating Cinnamaldehyde, Citral, Isoeugenol or Linalool on F. sporotrichioides Growth

**Figure 1:**
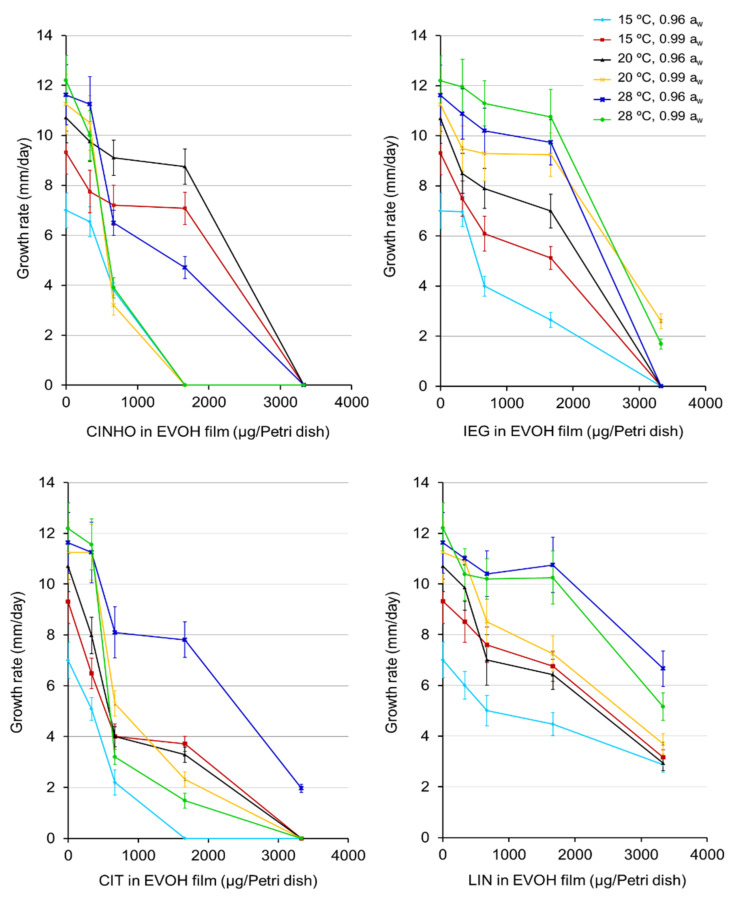
Growth rates (GR) of *F. sporotrichioides* in oat grain cultures incubated with EVOH films containing cinnamaldehyde (CINHO), citral (CIT), isoeugenol (IEG) or linalool (LIN) at different doses in vapor phase under different a_w_/temperature regimes. Incubation period: 21 days. Zero value on the *x*-axis corresponds to control cultures. Error bars represent standard deviations.

Circular colonies of *F. sporotrichioides* were observed on oat grains cultures under all conditions when growth occurred. Growth delay (time required for the colony to reach a size of 5 mm in diameter from the initial inoculum) was 2‒3 days depending on the temperature, a_w_, and treatment. The GR of *F. sporotrichioides* on oat grains in control cultures and treatments under the assayed conditions are shown in Figure 1. In control cultures without any film or with films without EO components, the development of fungal colonies under the same conditions was similar. Therefore, the values observed for control cultures in Figure 1 lie on the *y*-axis and correspond to the zero dose of the active compounds; they are the average of six replicates (three replicates × two repetitions) as no significant differences between the two repetitions were found. GR data were treated by ANOVA. Overall, GR in controls and treatments increased with increasing temperature in the order 15 °C < 20 °C < 28 °C (*p*-value < 0.0001), regardless of the a_w_ level. The post-hoc Duncan’s multiple range test showed three homogeneous non-overlapping groups with regard to temperature (Supplementary Table S1). However, in the case of CINHO treatments, growth at 28 °C was delayed with respect to lower temperatures (20 and 15 °C) at doses >333 µg/Petri dish. Although fungal colonies developed faster in cultures with 0.99 a_w_ than with 0.96 a_w_, multifactor ANOVA did not reveal significant differences concerning a_w_; the interactions of a_w_ × film type (*p*-value < 0.034) and a_w_ × temperature significantly affected fungal GR (*p*-value < 0.0006). The interactions a_w_ × temperature × film type and a_w_ × temperature × dose were also significant. Such interactions can influence the significance of particular main effects.

Different response profiles of *F. sporotrichioides* were observed concerning the type of bioactive film. Fungal GR was significantly affected by this factor (*p*-value < 0.001). According to the active compound the order of film effectivity was EVOH-CIT ≈ EVOH-CINHO > EVOH-IEG > EVOH-LIN. The Duncan’s test confirmed these results (Supplementary Table S1). With few exceptions, GR also decreased with increasing dose of the active compound regardless of the film type, a_w_ and temperature. With regard to dose, the Duncan’s test established four homogeneous groups without overlapping for doses 333, 666, 1665, and 3330 µg/fungal culture (25 g of oat grains), respectively (Supplementary Table S1). The dose × film type and temperature × film type interactions were also significant (*p*-value < 0.05).

The response of *F. sporotrichioides* growth to the treatments (Figure 1) was used to calculate the effective doses of each component in films to reduce GR by 50%, 90%, and 100% (ED_50_, ED_90_ and ED_100_) under the assayed conditions (Table 1). Doses higher than 3330 µg of active ingredient per plate were not tested as they were considered of less interest in food matrices. Although plant EOs or their individual components are very volatile, and hence easily removed from the food once it comes into contact with the environment, high concentrations can affect food organoleptic properties due to their intense aroma.

As shown in Table 1, the ED_50_ in oat cultures of *F. sporotrichioides* for EVOH films containing CINHO, IEG, CIT and LIN were in the ranges 545–2310, 1030–2585, 515–2235, 2175–> 3330, respectively. With few exceptions, ED_90_ and ED_100_ could be estimated for EVOH-CIT, EVOH-CINHO and EVOH-IEG and generally they were in the range of the highest tested doses (1665 to 3330 µg/Petri dish). ED_90_ and ED_100_ values for EVOH-LIN always exceeded the highest assayed dose.

### 2.2. Effect of EVOH Films Incorporating Cinnamaldehyde, Citral, Isoeugenol or Linalool on T-2 and HT-2 Production by F. sporotrichioides

**Figure 2:**
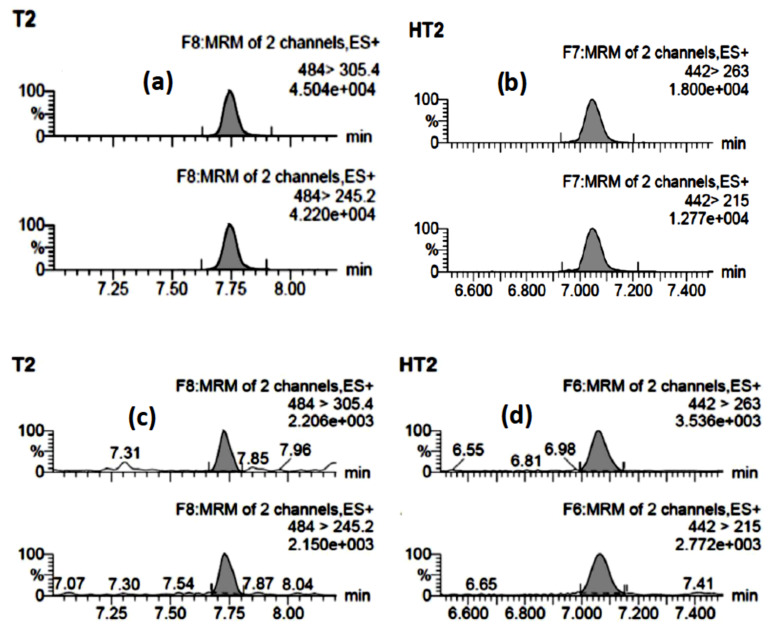
UPLC-(ESI+)-MS/MS MRM chromatograms of T-2 and HT-2: (**a**) standard solution of T-2 (90 ng/mL); (**b**) standard solution of HT-2 (75 ng/mL); (**c**) T-2 in an extract from a culture of *F. sporotrichioides* on oat grain; (**d**) HT-2 in an extract from a culture of *F. sporotrichioides* on oat grain; for each mycotoxin, the upper chromatogram corresponds to the quantifier ion (Q) and the lower one corresponds to the qualifier ion (q). Notation note: *y*e + 00*x* means *y* × 10*^x^*.

The analytical methodology for determination of T-2 and HT-2 concentrations in the cultures was validated by determination of the limits of detection (LOD) and quantification (LOQ), the method accuracy, by means of recovery assays of blank oat grain spiked at different concentrations, and the precision by repeatability assays made on spiked oat grain. The results are summarized in Table 2. UPLC-MS/MS MRM chromatograms of T-2 and HT-2 in standard solutions and extracts of cultures of *F. sporotrichioides* grown on oat grain appear in Figure 2.

**Figure 3:**
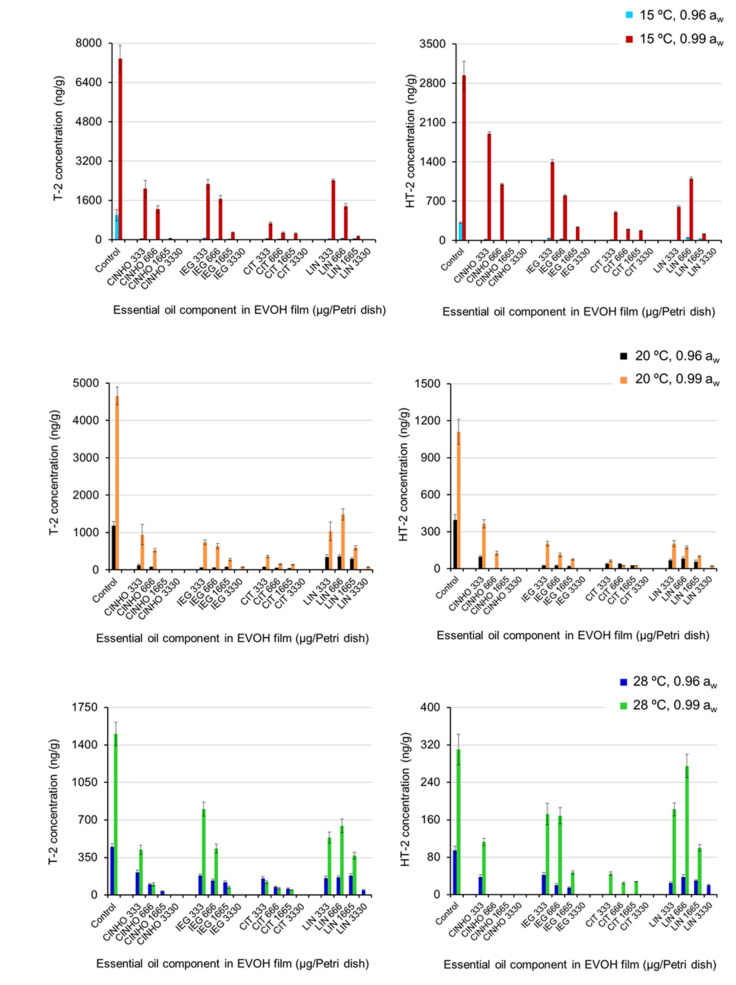
Production of T-2 and HT-2 by *F. sporotrichioides* in oat grain cultures incubated with EVOH films containing CINHO, CIT, IEG or LIN at different doses in vapor phase under different a_w_/temperature regimes. Incubation period: 21 days. Error bars represent standard deviations.

Cultures of *F. sporotrichioides* under all the assayed conditions were examined to determine T-2 and HT-2 production. Figure 3 shows T-2 and HT-2 mean levels in controls and treatments. In control cultures, the maximum T-2 and HT-2 mean levels (7.37 × 10^3^ ng/g and 2.94 × 10^3^ ng/g, respectively) were found at 0.99 a_w_ and 15 °C and the minimum T-2 and HT-2 mean levels (448 ng/g and 95 ng/g, respectively) were found at 0.96 a_w_ and 28 °C. In general, the level of both toxins was significantly lower in cultures with 0.96 a_w_ than with 0.99 a_w_ (*p*-value < 0.0001) and was also significantly lower in treated cultures than in controls (*p*-value < 0.0001). In the case of EVOH-CINHO according to the Duncan’s test, the lowest dose did not significantly reduce HT-2 production with respect to controls taking into account all factors dose/temperature/a_w_. However, a pairwise comparison of means of the lowest dose (leaving out the remaining doses) and controls indicated the existence of a significant difference between them.

A significant influence (*p*-value < 0.005) of temperature on the reduction/inhibition of the biosynthesis of both toxins was also found. For temperature, the Duncan’s test arranged three homogeneous non-overlapping groups (Supplementary Table S1). The first corresponded to the cultures incubated at 15 °C (high toxins levels), the second, to those incubated at 20 °C (intermediate toxin levels), and the third to those incubated at 28 °C (low toxins levels). In the case of HT-2, the post-hoc Duncan’s test placed the cultures incubated at 15 °C in one homogeneous group, and those incubated at 20 and 28 °C in another one (Supplementary Table S1).

The type of film also significantly affected T-2 and HT-2 production. Concerning T-2 level, the Duncan’s test (Supplementary Table S1) established three homogeneous although overlapping groups: one group for treatments with EVOH-CIT film (the most effective in inhibiting toxin biosynthesis), a second group formed by EVOH-CINHO and EVOH-IEG films, and a third group for EVOH-IEG and EVOH-LIN. However, for HT-2, although the film type did not significantly affect HT-2 production (*p*-value = 0.077), toxin levels were generally lower in treatments with EVOH-CIT films than in the rest of treatments.

The dose also had a significant effect on T-2 and HT-2 production. For T-2 toxin, the Duncan’s test distinguished one homogeneous group for each dose, regardless of film type. However, for HT-2, this test distinguished two groups, one for the films containing the two major doses of active compounds (1665 and 3330 μg/plate) and another group for the films with the two minor doses (333 and 666 μg/plate) (Supplementary Table S1). With few exceptions, when the dose of active compound in the film increased, the production of both toxins decreased. Likewise, in general, doses of active compound in EVOH-CINHO, EVOH-CIT, and EVOH-IEG films between 1665 and 3330 μg/plate totally inhibited T-2 and HT-2 production, depending on temperature and a_w_.

In addition to the individual factors mentioned above, the following interactions had a significant influence on the reduction/inhibition of T-2 biosynthesis: a_w_ × temperature, a_w_ × dose, a_w_ × film type, dose × film type, dose × temperature, a_w_ × dose × temperature and a_w_ × dose × film type (*p*-value < 0.02). For HT-2, significant interactions were a_w_ × temperature, a_w_ × dose, dose × temperature, and a_w_ × dose × temperature (*p*-value < 0.001).

### 2.3. Prediction of F. sporotrichioides GR and T-2 and HT-2 Production Using ML Algorithms

**Figure 4:**
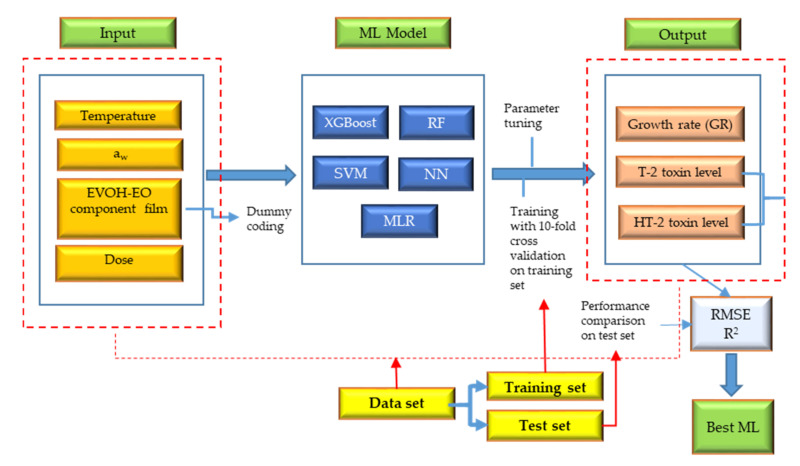
Framework of ML algorithm design and comparison for predicting growth rate and accumulation of T-2 and HT-2 toxins in cultures of *F. sporotrichioides* incubated on oat grain.

**Figure 5:**
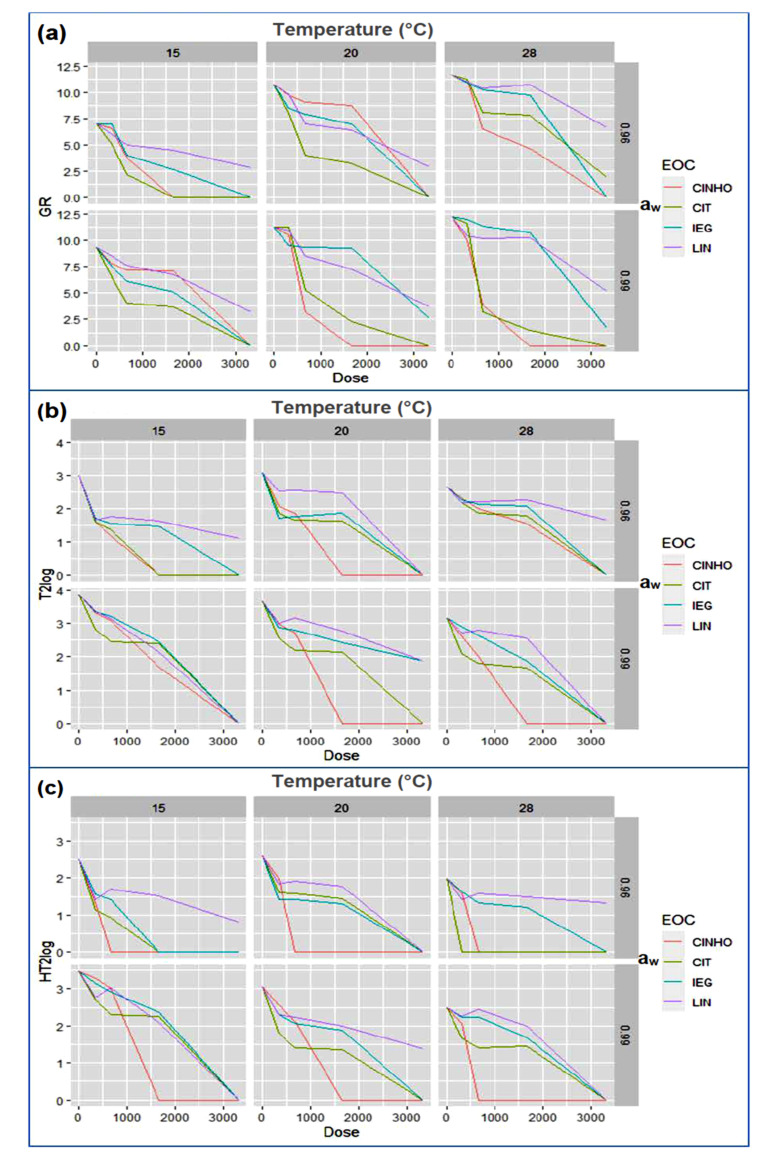
Graphs of the datasets for GR (**a**), T-2 level (**b**), and HT-2 level (**c**). The input variables are: temperature (top) with three levels (15, 20 and 28 °C), a_w_ (right) with two levels (0.96 and 0.99), essential oil component (EOC) of the EVOH films (color lines connecting data points) with four levels (CINHO, CIT, IEG and LIN), and dose of the essential oil component (bottom) with five levels (0, 333, 666, 1665 and 3330 µg/culture). The output variables are on the left side of the Figure: GR is measured in mm/day; for both T-2 and HT-2 toxins the output values are the decimal logarithms of their concentrations plus 1. Only mean values are considered for inputs and outputs.

The XGBoost, RF, SVM, and NN models were trained and validated using 10-fold cross-validation on the training dataset which was randomly chosen from the whole data set. The scheme of the process framework can be seen in Figure 4. The dataset is graphically depicted in Figure 5.

The variation of the root mean square error (RMSE) for various ML algorithms during the training/validation processes for predicting GR can be observed in Supplementary Figure S1. The RMSE variation for T-2 and HT-2 with different algorithms appear in Supplementary Figure S2. The coefficients of MLR equations were estimated by the least square method and the data used for this process was the same used for training/validating the remaining algorithms. The features of the best ML models found and their RMSE and R^2^ (coefficient of determination) values applied to the test dataset appear in Table 3. Five models (NN, RF, SVM, XGBoost, and MLR) were tested on the same data set for each output variable (GR, and levels of T-2 and HT-2 toxins). The test set was not used during training/validation or adjusting by least square method (MLR). The optimized parameters for each ML model are in the third column of Table 3.

RF always used the maximum number of selected predictor variables (mtry = 4) to provide the minimum RMSE and SVM always used the same value for the regularization parameter (C = 1) to get the best performance. Model performance increases when the RMSE (the selected metrics) reaches a minimum value and when the R^2^ value increases approaching to the unit. The models giving the lowest RMSE values usually provided the maximum R^2^ values (Table 3).

The intercept and six coefficients obtained for the MLR models to predict GR, and levels of T-2 and HT-2 and their significance are listed in Supplementary Table S3. The coefficients for the input variable dose were highly significant for all outputs. Those for a_w_ were highly significant for T-2 and HT-2 toxins, but this coefficient was non-significant for GR. The coefficient for temperature was highly significant for GR but not for both mycotoxins. The coefficient for EVOH-LIN was highly significant for all the outputs while that for EVOH-IEG was less significant and the third coefficient (EVOH-CIT) was not significant. The coefficient for EVOH-CINHO is not shown because film type is a categorical variable with four levels and this was the omitted level (taken as the reference level) by the software during dummy variable coding (see Section 5.7.2).

For GR prediction, the XGBoost algorithm produced the best results (RMSE = 1.16 mm/day; R^2^ = 0.919 for the test set) (these are the lowest RMSE and the highest R^2^ value), followed by RF and NN. For the prediction of T-2 production, RF provided the minimum RMSE and the maximum R^2^ value, followed by XGBoost and NN. For HT-2, the best performance was offered by XGBoost, as in the case of GR, followed by RF and SVM. MLR gave the maximum RMSE resulting in the worst model. The NN did not work well with HT-2.

The scatter plots (predicted versus observed output values) together with the lines of best fit (obtained by linear regression of predicted against observed output values) for all the built ML models on the test sets are shown in Supplementary Figure S3 (for GR), and Supplementary Figure S4 and Supplementary Figure S5 for T-2 and HT-2, respectively. For every point on the scatter plots the X-score is the observed output value. The Y-score of blue points is the output value predicted by the model; for the points on the best fit line (red points) the Y-score is the ideal predicted output value by the model if there were a linear relationship between Y and X. The differences between both Y coordinates are the residuals. The ideal line of perfect fit is Y = X (slope = 1, intercept = 0) in which prediction errors do not exist, thus becoming RMSE = 0 and R^2^ = 1. The lines of best fit for XGBoost and NN to model GR in the test set statistically match (*p*-value = 0.05) the respective lines of perfect fit, but the first one has less residual variance and the origin approaches more to zero (Supplementary Figure S3). It also happens with XGBoost and RF for predicting HT-2 toxin in the test set (Supplementary Figure S5). For T-2, the slope of the best fit line associated to the XGBoost model was statistically the unit, but the intercept was not zero (Supplementary Figure S4).

## 3. Discussion

The biggest problem of the possible application of EOs or pure components of EOs on the control of toxigenic fungi and mycotoxin in foods is their high volatility and intense aroma. Therefore, it is necessary to know the minimum effective doses to reduce/inhibit fungal growth and mycotoxin biosynthesis and to design strategies to achieve their slow and prolonged release on the food surface. One of the strategies and perhaps the most interesting one is the inclusion of EOs or their pure components in packaging systems such as films. Among them, EVOH films have given good results against mycotoxin-producing fungi and on the reduction or total inhibition of ochratoxin A, aflatoxins, fumonisins or zearalenone production [37,38,39]. However, according to these studies, the response of toxigenic fungi to EOs or their pure components may vary depending on the fungal species, substrate and environmental conditions. Therefore, to select the most effective EO component, encompassing the broadest spectrum of action, more investigations with different mycotoxin-producing species that coexist in the same substrate should be developed. Moreover, in preventive treatments, it is crucial to have good prediction tools, such as those based on ML algorithms that allow determining a priori the recommended treatment in each case. The present study focuses on the design and testing of four types of films containing CINHO, IEG, CIT, and LIN at different doses against *F. sporotrichioides* and T-2 and HT-2 production in oat grain. In addition, considering the importance of temperature and a_w_ of the substrate in fungal growth and mycotoxin production, the ED_50_, ED_90_, and ED_100_ for each bioactive film were determined under all the assayed conditions. The interactive effect of all these parameters is studied and different ML algorithms were applied and their performance has been compared. Oat grain has been chosen as matrix because oats represent the most contaminated cereal with T-2 and HT-2 and the most favorable substrate for their production [54].

In the present study, GR of *F. sporotrichioides* decreased with decreasing temperature following the order 28 °C > 20 °C > 15 °C and high tolerance of the fungus to a_w_ levels (0.99 and 0.96) was observed. In contrast, the production patterns of T-2 and HT-2 were different. Generally, T-2 and HT-2 levels in cultures varied with temperature and a_w_ following the order 15 °C > 20 °C > 28 °C and 0.99 a_w_ > 0.96 a_w_. These results agree with those reported by Nazari et al. [21]. They found optimal growth of *F. sporotrichioides* at 25–30 °C and optimal sporulation at 32.3 ± 2.1 °C. Likewise, in assays with three strains of *F. sporotrichioides* in an oat-based medium similar results were found [55]. A decrease in toxin production in *F. sporotrichioides* cultures was also found when water stress increased [56]. However, for *F. langsethiae,* it has been reported that the optimal temperature for sporulation is 24.5 ± 0.7 °C [21] and the optimal temperature for growth and T-2 and HT-2 production is 25 °C [4,19,20,55]. This species is less permissive to a_w_ stress than *F. sporotrichioides* [55]. Therefore, considering our results and those previously reported, it can be concluded that the optimal temperature for growth of these two closely related species could differ by 2–3 °C, and for T-2 and HT-2 production by 10–12 °C*,* regardless of the strain, resulting *F. sporotrichioides* more tolerant to a_w_ stress than *F. langsethiae.* This fungal response could partly explain the prevalence of *F. sporotrichioides* in southern European regions and the lower incidence of T-2 and HT-2 in cereals grown in these countries than in central and northern European regions.

A comparative analysis of the results obtained in cultures of *F. sporotrichioides* treated with bioactive films with previous reports is difficult. Knowledge about the effect of EOs against *F. sporotrichioides* is extraordinarily scarce. In the present study, the effectiveness of the EO components included in EVOH films to control *F. sporotrichioides* growth was in the order CIT ≈ CINHO > IEG > LIN and in the control of T-2 toxin production the efficacy followed the order CIT > CINHO ≈ IEG > LIN. It has been reported that in YES-agar amended with 100 μM of a form of CINHO (trans-cinnamaldehyde thiosemicarbazone), mycelial growth of *F. sporotrichioides* and mycotoxin production were inhibited by 24.9% and 91.4%, respectively [57]. Metabolic changes in *Euphorbia palustris* latex after infection with *F. sporotrichioides* have been observed. The latex of infected plants was found to contain higher levels of benzoyl ingenol-laurate and 24-methylenecycloartanol, and these compounds were strongly correlated with the antifungal activity of the latex [58]. In contrast, the antifungal action of EOs or active component of EOs against phytopathogenic and/or toxigenic *Fusarium* spp. apart from *F. sporotrichioides* has been widely tested in aqueous solution by different methods, such as inclusion within the culture medium or disk diffusion. Among numerous EOs tested, some have been selected for their high effectiveness against certain *Fusarium* spp. Thus, the minimal inhibitory concentrations (MIC) of clove, lemon grass, mint, and eucalyptus EOs against *F. oxysporum* f. sp. *lycopersici* were 31.25, 62.5, 125, and 500 ppm, respectively [59]. Clove and thyme oils, pure citral, eugenol and thymol at 500 μL/L, reduce growth of *F. oxysporum* colonies and total inhibition of all isolates was observed when the EOs were used at a concentration of 1000 μL/L [60]. The MICs of cinnamon oil, natural cinnamaldehyde, and synthetic cinnamaldehyde against *F. verticillioides* were 60, 50, and 45 mL/L, respectively [61]. It was also found that EOs extracted from cumin, have antifungal activity against various *Fusarium* spp. The diameter of the fungal growth inhibition zone ranged 2.17–0.00 cm depending on the fungal species and doses [62]. The oil of *Ocimum gratissimum* rich in eugenol has high effectivity against *F. verticillioides* and induces an inhibitory effect on FB1 production [63]. Likewise, thyme oil is very effective against *F. verticillioides* [64]. Low concentrations (50–100 μg/g) of different EOs were effective to control growth of *F. culmorum* in synthetic medium but concentrations of 500 μg/g were required to control fungal growth on sterile wheat grains [65]. Therefore, the class of substrate is an important factor to take into account when conducting comparative studies.

Despite the different methodology used, a comparative analysis of the results obtained in the present study with previously reported results with different EOs and *Fusarium* species shows that CINHO, CIT, and IEG are among the most effective EO components against toxigenic *Fusarium* spp. and mycotoxin production, and in particular against *F. sporotrichioides* and the biosynthesis of T-2 and HT-2 in oats. Although in the cited previous studies, the active compounds were not included into EVOH films and, in general, the assays were made in synthetic media, all these reports provide interesting information about the EOs or EO components that could offer better results in the preparation of packaging systems, paints, coatings or other active materials against toxigenic *Fusarium* spp. that frequently cohabit in foods, such as cereals or cereal products during their silage, transport, or marketing. However, future research with a wider variety of EOs or pure components of EOs against *F. sporotrichioides* and the production of T-2 and HT-2 toxins will be necessary.

As far as we know, the effect of bioactive films containing EOs or their pure components on the growth of *F. sporotrichioides* and on T-2 and HT-2 toxin production has not been studied to date. However, in recent studies carried out with relevant toxigenic *Aspergillus* spp. [37,38] and *Fusarium* spp. [39], using EVOH films containing EOs or their active components, similar results to those obtained in the present study were recorded. Thus, in cultures of *A. flavus* and *A. parasiticus* on maize grains, the order of efficacy of EVOH-EO films to control fungal growth and aflatoxin production was EVOH-CINHO > EVOH-carvacrol > EVOH-*Origanum vulgare* EO > EVOH-*Cinnamomum zeylanicum* EO. For the most bioactive films (EVOH-CINHO) the ED_50_, ED_90_, and ED_100_ to reduce/inhibit *A. flavus* and *A. parasiticus* growth *(*both species associated with a strong production of aflatoxins) were in the ranges 121–133, 222–229, and 250 μg active compound/fungal culture (25 g maize kernels), respectively, depending on environmental conditions [37]. In assays against *A. tubingensis* and *A. steynii*, the effectiveness of the films followed the order EVOH-CINHO > EVOH-IEG > EVOH-CIT > EVOH-LIN. The ED_50_, ED_90_, and ED_100_ of the best bioactive film (EVOH-CINHO) against growth of both species were in the ranges 165–405, 297–614, and 666 μg active compound/fungal culture (25 g maize kernels), respectively, depending on environmental conditions [38]. In the same way, bioactive EVOH films incorporating CINHO, CIT, IEG, or LIN have been assayed against *F. culmorum* (a species able to produce high levels of zearalenone) and against *F. proliferatum* (a species able to produce high levels of fumonisins B1 and B2). The efficacy of the films was in the order EVOH-CIT *>* EVOH-CINHO *>* EVOH-IEG *>* EVOH-LIN. ED_50_, ED_90_, and ED_100_ of CIT in EVOH films against both species were in the ranges 200–490, 450–630, and 660 μg active compound/fungal culture (25 g maize kernels), depending on the environmental conditions [39]. In the present study, the order of effectiveness of the EVOH films against *F. sporotrichioides* (one of the best producing species of T-2 and HT-2 toxins) was EVOH-CIT > EVOH-CINHO > EVOH-IEG > EVOH-LIN, as described in [39]. Therefore, the most effective film was EVOH-CIT and its ED_50_, ED_90_, and ED_100_ were in the ranges 515–2235, 1350–>3330, and 1665–>3330 μg active compound/fungal culture (25 g oat grains) depending on temperature and a_w_. In general, with few exceptions, it was observed that a decrease or total inhibition of the fungal growth was parallel to a reduction or total inhibition of mycotoxin biosynthesis, regardless of the species, type of films, dose, and environmental conditions.

The comparative analysis of the results obtained in the present study and in previous reports indicates that the tested EVOH films containing CIT, CINHO or IEG, at acceptable doses of the active principle, are effective in the simultaneous control of the growth of relevant toxigenic *Fusarium* spp. and *Aspergillus* spp. They can also be very effective tools in reducing/inhibiting the production of the main mycotoxins associated with these species and they can be considered as potential strategies in the management of both risks, fungal growth and mycotoxin production, in foods. EVOH films containing bioactive compounds, such as EOs, could be used as coating of sealed plastic bags avoiding or delaying fungal growth and mycotoxin production on storage and transportation. EVOH copolymers have great potential as vehicles for the manufacture of antimicrobial AP materials. These active materials have been successfully prepared by extrusion and by coating technologies [66]. In addition, oats are richer in fat (7%) than other cereals, such as wheat, barley, maize, or rice (1.5–2%) and more prone to suffer lipid oxidation during storage, which shortens shelf life. Thus, the storage of properly dried cereal grain in sealed bags can be very useful to avoid deterioration by fungal growth [41,42]. These AP materials may also be useful in the packaging of derivatives of oats and other cereals such as bread [40,67,68].

As there are very few reports concerning ML applications in the field of predictive mycology a comparative analysis of their application to predict fungal growth and mycotoxin production is difficult. This is the first attempt to apply ML models to the prediction of *F. sporotrichioides* growth and T-2 and HT-2 production.

Regarding the efficacy of the tested ML algorithms to predict GR by this species on oat grain cultures with EVOH films containing CINHO, CIT, IEG, and LIN, XGBoost produced the best results although this algorithm presents the greatest complexity in computation tasks. The following models in terms of best accuracy were RF and NN; SVM was fourth and the cost function stopped improving for regularization values C > 1, while the regression model showed the worst performance. Notwithstanding, small differences in the RMSE are not very relevant to choose the best model, taking into consideration that output values have always an uncertainty owing to their experimental nature.

The comparison of ML models is a useful task to obtain the best technique to accurately predict or classify outcomes in diverse fields of the knowledge on the basis of known features. After a comparison among different ML models, it was concluded that the XGBoost model has obvious advantages of performance compared to the other tested ML models. In the literature, it has been successfully used to predict mortality of patients with acute kidney injury in intensive care unit [69]. Moreover, after comparison with ML algorithms, e.g., logistic regression (LR), kNN, decision tree (DT), SVM, and RF, the XGBoost algorithm combined with clinical information, laboratory tests, and other features, aimed at predicting the possibility of COVID-19 patients becoming severe and critically ill, provided excellent performance [70]. In another study, various ML algorithms (SVM, NN, RF, DT, LR, and kNN) have been compared to predict the mortality rate in patients with COVID-19 using 10-fold cross-validation; all models provided accurate results in the range of 86.87–89.98%, although the NN ranked first, followed by kNN, SVM and RF [71]. However, in the field of application of ML in predictive mycology and prediction of mycotoxin accumulation in food or feed the number of published papers is quite low [39,47,48,49,50,53]. Two deep NN models were trained to predict, at harvest, which maize fields were contaminated beyond the legal limit with aflatoxin B1 and fumonisins reaching an accuracy >75% demonstrating the ML approach added value with respect to classical statistical approaches such as simple regression or MLR models [72]. A comparative study of NN, RF, and XGBoost models to predict the growth of *F. culmorum* and *F. proliferatum* and production of zearalenone and fumonisins in treatments with different formulations of three chemical fungicides at two temperatures and two a_w_-values in maize extract medium was carried out by our group. The XGBoost model provided the best results [53]. In another study using bioactive EVOH films containing the same EO active components employed in the present study, the RF algorithm encompassing the same parameters (ntree = 500, mtry = 4) produced the best models (lowest RMSE for the test set) for predicting GR and zearalenone by *F. culmorum* or GR and fumonisin production by *F. proliferatum* on partially milled maize kernels [39]. MLR provided the worst results, as in the present study. The application of ML to predict the GR of toxigenic fungal species and production of mycotoxins in food matrices is expected to be greatly implemented in future studies as the built models have been shown to be useful tools for this task. Further improvements should include more levels of the input variables and the implementation of different input variables, such as kind of substrate/food commodity in which the fungi grow and its treatment, atmosphere composition, incubation time, amount of inoculum, etc.

## 4. Conclusions

The present study allow us to draw some general conclusions: (1) *F. sporotrichioides is* more resistant to the tested treatments (EVOH films containing EOs or pure components of EOs) than previously studied toxigenic species, such as *F. culmorum, F. proliferatum, A. steynii*, *A. tubingensis, A. flavus* and *A. parasiticus (*the most sensitive), depending on temperature and a_w_. (2) EVOH-CIT, EVOH-CINHO, and EVOH-IEG could be very valuable tools in the reduction or total inhibition of the growth of all these relevant toxigenic *Fusarium* spp. and *Aspergillus* spp., which frequently cohabit in foods, such as cereals and cereal derivatives. (3) In general, the reduction or total inhibition of T-2 and HT-2 production in the treatments of *F. sporotrichioides* was a direct consequence of the reduction or total inhibition of fungal growth. This correlation between fungal growth/mycotoxin level in treatments with EVOH films containing EOs or pure components of EOs, on grain cereals, was previously observed for *F. culmorum*/zearalenone, *F. proliferatum*/fumonisins B1 and B2, *A. steynii*/ochratoxin A, *A. flavus*/aflatoxins B1 and B2, and *A. parasiticus*/aflatoxins B1, B2, G1, and G2. (4) Temperature and a_w_ can significantly affect the effective doses (ED_50_, ED_90_ and ED_100_) of EO components included in EVOH films in treatments against fungal growth and mycotoxin production. For this, these physicochemical factors, and other possible parameters, should be considered, in practice, as possible complementary treatments. Since the interactions between all these parameters can significantly reduce the effective dose of EOs or pure components of EOs included in the EVOH film. (5) Five ML predictive models have been developed and their performance has been compared to find the best model able to predict as accurately as possible GR and production of T-2 and HT-2 in cultures of *F. sporotrichioides* on oat grain provided with EVOH films containing EO components (in vapor phase) at different doses under diverse combinations of temperature and a_w_. The best ML model for predicting GR and HT-2 toxin production were built with the XGBoost algorithm followed by the RF, while for T-2 toxin the RF model provided the best results, followed by XGBoost. Hence, these two algorithms can be used to make the most accurate predictions of the studied outcomes. The linear regression model (MLR) proved to be the worst while NN and SVM ranked either third or fourth. Further work is needed to extend the application of these active EVOH films containing EO components to areas of food protection like food packaging to take advantage not only of the physicochemical properties of these films but also of the antioxidant and antimicrobial properties of the added EO compounds. Food products sealed in packages made of these bioactive films will be protected against the proliferation of fungi and other microorganisms, and hence against production of mycotoxins during storage, which will contribute to increasing food shelf life and safety for consumers. Future research is needed to establish the stability of the EO components in the polymer with time. Furthermore, the standardization of EOs in a way that ensures their use without alterability is a relevant issue. Another possible way to expand this and related studies in the addition of different EO components or a mix of two different components to EVOH films and to assay its activity against a variety of toxigenic fungi. In the long term, due to the awareness that the use of non-recyclable/non-compostable plastics has awakened in consumers and authorities, it is expected that there will be an increased variety of new bioplastics made from non-fossil materials and they will replace, at least in part, the plastic films commonly used at present. Then, the addition of EOs or their active components to these bioplastics should be considered and studied in-depth. The application of ML models to all of these possibilities in order to make accurate predictions of the outcomes is expected to be increased in the near future.

## 5. Materials and Methods

### 5.1. Film Preparation

Ethylene-vinyl alcohol copolymer with 29% ethylene molar content (EVOH-29) was supplied by The Nippon Synthetic Chemical Industry Co., Ltd. (Osaka, Japan). *Trans*-cinnamaldehyde (3-phenyl-2-propenal) (CINHO), linalool (LIN), isoeugenol (IEG), and citral (CIT) were supplied by Sigma-Aldrich (Barcelona, Spain). The preparation of the EVOH films was carried out as previously described [37]. The final content of the EO components was determined by GC [39,73]. The films were labeled as EVOH-CINHO, EVOH-CIT, EVOH-IEG, or EVOH-LIN. The final EO component contents for the prepared films and their grammage was previously reported [39].

### 5.2. Standards and Reagents

Standards of T-2 and HT-2 toxins were supplied by Sigma (Sigma–Aldrich, Alcobendas, Spain). Formic acid was purchased from Panreac (Castellar del Valles, Spain). Ammonium formate (>99% for LC-MS) was from VWR International Eurolab, S.L. (Llinars del Vallés, Spain). Acetonitrile (ACN) and methanol (LC grade) were purchased from J.T. Baker (Deventer, the Netherlands). Pure water was delivered from a Milli-Q apparatus (Millipore, Billerica, MA, USA).

### 5.3. Inoculum Preparation

*F. sporotrichioides* strain MN861801 (NCBI) was used. The strain is held in the Mycology and Mycotoxins Group Culture Collection (Valencia University, Spain). The strain was grown on Petri dishes containing sterilized maize extract medium (3% *w/v* of ground maize kernels + 2% *w/v* agar in pure water). Five μL of a conidia suspension (1.0 × 10^6^ conidia/mL) of the species was inoculated on the center of plates and incubated at 28 °C for 7 days. A suspension of spores containing 1.0 × 10^6^ conidia/mL was prepared before each experiment [39].

### 5.4. Preparation of Fungal Cultures with Different EVOH Films and a_w_ Levels

The previously indicated methodology [39] was followed with some differences. Briefly, the assays were performed in 25 g autoclaved oat kernels containing undetectable levels of T-2 and HT-2. Oat grain a_w_ was adjusted to 0.99 or 0.96. The sterilized grains were spread into sterile Petri dishes and circular pieces of each bioactive EVOH film were stuck on the inner part of the lid. The final doses of each component were calculated [39]. Culture media were inoculated with the *F. sporotrichioides* strain, capped, and sealed with Parafilm M^®^. The Petri dishes undergoing the same treatment were enclosed in sealed plastic containers together with beakers of a glycerol–water solution matching the same a_w_ as the treatments. Treatments and control cultures were incubated at 15, 20, and 28 °C for 21 days in the darkness. Experiments were run in triplicate and repeated twice.

### 5.5. Effect of Treatments on Fungal Growth

Colony growth in cultures was examined daily during the incubation period by measuring two mutually perpendicular colony diameters with the help of a magnifying glass. Mean colony diameter was plotted against time. GR was calculated as the slope of the line obtained by linear regression of mean colony diameter against time, using only the linear part of the plot. The plots of GR versus dose were used to estimate the ED_50_, ED_90_, and ED_100_ of the EO components, when possible [39].

### 5.6. Effect of Treatments on Production of T-2 and HT-2 Toxins

#### 5.6.1. Mycotoxin Determination in Oat Cultures

The previously indicated methodology [39,53] was followed with little changes bearing in mind the matrix nature and the mycotoxins to be determined. Briefly, after 21 days, cultures were removed from dishes, dried, milled, homogenized and analyzed for T-2 and HT-2. Two g of culture was extracted with 8 mL of ACN/water/formic acid (80/19/1, *v/v/v*). After centrifugation, a portion of the supernatant was filtered (0.22-μm PTFE) and injected into the UPLC system. Extracts containing very high mycotoxin levels were diluted with the same solvent mixture and re-injected. Matrix-matched calibration was performed. Standard solutions of T-2 and HT-2 were prepared by the dissolution of standards in ACN/water (1/1, *v/v*) and further dilution with acetonitrile/water/formic acid (80/19/1, *v/v/v*). Calibration standards were prepared covering the ranges 3–600 ng/mL for T-2 and 1.2–600 ng/mL for HT-2. Calibration equations were obtained by weighted linear regression of areas versus concentrations. Validation was carried out by replicated recovery assays [39]. The concentration ranges in spiked oat blanks were 12–120 ng/g for T-2, and 4.7–47 ng/g for HT-2.

#### 5.6.2. UPLC-MS/MS Conditions

The conditions for the UPLC separation and MS/MS detection have been reported [74]. Two product ions (one quantifier, and other assistant qualifier) were monitored for each mycotoxin. Retention time of the target peaks in multiple reaction monitoring (MRM) chromatograms was used for identification and the area of the quantifier peak was interpolated in the calibration line to achieve quantification. The MS/MS and ESI+ conditions as well as the transitions involved in the analysis of the two toxins are in Supplementary Table S2.

### 5.7. Statistics and Data Treatment

#### 5.7.1. Analysis of Variance

Data were analyzed by multifactor analysis of variance (ANOVA). Post-hoc Duncan’s multiple range test (α = 0.05) was used to find homogenous groups when ANOVA showed the existence of significant differences. Raw output values were transformed as log_10_(x + 1) where x was the variable value to reduce spread. The software package used was Statgraphics Centurion XV.II (StatPoint, Inc., Herndon, VA, USA).

#### 5.7.2. ML Models Tested

Several regression models were comparatively tested using the same data set for modeling GR and T-2 and HT-2 production in oat cultures of *F. sporotrichioides*. They were artificial neural networks (NN) (neuralnet library) [75], RF (randomForest library) [76], support vector machines (SVM) (SVM library), XGBoost (XGBTree library) [77], and multiple linear regression (MLR). The working methodology was previously described [39,53]. The software used was R and the ‘classification and regression training’ (caret) package [78]. SVM is a binary classification method that separates two classes by a linear boundary and relies on extended linearity. In SVM, the main goal is to find the optimal separation hyperplane between two classes in a higher dimensional space, leading to more accurate classification and a reduction in generalization error.

The input variables were temperature, a_w_, film type, and dose of EO component in the EVOH films. The output variables were GR, T-2 concentration, and HT-2 concentration. The output values were averaged as only one output value is associated with a set of input values. Variables were preprocessed before mathematical treatment. For mycotoxin outputs, the decimal logarithm of average mycotoxin concentration plus 1 was computed. The categorical input variable (type of EVOH film) was transformed in numerical variables by recoding them as dummy variables [39]. An easy explanation of this coding procedure can be read in [79]. Before training the ML models, every data set was randomly split into training (approximately 75%) and test (approximately 25%) set. The training set was used to build/cross-validate the ML models and the test set was used to assess its performance on independent data. MLR uses all independent variables to predict the output value and the coefficients are chosen by the least squares method. It is assumed that there is a linear relationship between the dependent variable and independent variables.

The metric used for obtaining the best parameters (Table 4) and for performance evaluation of the ML algorithms was the RMSE over 10-fold cross-validation, a resampling procedure used to evaluate ML models on a limited data sample. The RMSE is measured in the same units as the observed/predicted values and can be severely affected by large error values. The coefficient of determination or R-squared (R^2^), which is used to evaluate how much of the variation in outputs can be explained by the variation in the input variables, was also obtained.

The ML models were tuned to optimize their performance (minimum RMSE) for each task, according to their most significant parameters (Table 4). The best predictive ML models obtained after training/cross-validation (minimum RMSE for validation) or by the least square method, in the case of MLR, were further tested and compared against a test set (28 output values, approximately 25% of the dataset), which was not used in the training/10-fold cross-validation task of the models. The calculated RMSE and R^2^ values for the test sets provide an insight regarding the model performance serving as a comparison tool to choose the best model.

## Figures and Tables

**Table 1:** Effective doses (ED_50_, ED_90_ and ED_100_) (µg/Petri dish) of pure components of essential oils in EVOH films to control growth of *F. sporotrichioides* on oat grain under different conditions of temperature and a_w_. CINHO: cinnamaldehyde; IEG: isoeugenol; CIT: citral; LIN: linalool.

Temp. (°C)	a_w_	Individual Components of Essential Oils in EVOH Films											
		CINHO			IEG			CIT			LIN		
		ED_50_	ED_90_	ED_100_	ED_50_	ED_90_	ED_100_	ED_50_	ED_90_	ED_100_	ED_50_	ED_90_	ED_100_
15	0.96	745	1480	1665	1030	2890	3330	515	1350	1665	2680	>3330	>3330
	0.99	2240	3120	3330	1820	3035	3330	580	2910	3330	2640	>3330	>3330
20	0.96	2310	3130	3330	2065	3080	3330	555	2790	3330	2175	>3330	>3330
	0.99	555	1315	1665	2585	>3330	>3330	650	2525	3330	2430	>3330	>3330
28	0.96	1050	2920	3330	2340	3130	3330	2235	>3330	>3330	>3330	>3330	>3330
	0.99	545	1350	1665	2520	>3330	>3330	550	1975	3330	3025	>3330	>3330

**Table 2:** Mean retention times, limits of detection (LOD) and quantification (LOQ), mean percentage of recoveries and mean relative standard deviation of recoveries under conditions of repeatability (RSDr) for T-2 and HT-2 toxins produced by *F. sporotrichioides* in oat grain cultures.

Mycotoxin	Mean Retention Time (Min)	LOD (ng/g)	LOQ (ng/g)	Mean Recovery (%)	Mean RSDr
T-2 toxin	7.72	4.0	12	95.8	8.5
HT-2 toxin	7.04	1.5	4.6	97.4	4.7

**Table 3:** Best machine learning (ML) model performance for predicting GR and T-2 and HT-2 production by *F. sporotrichioides* cultured on oats grains with EVOH films containing CINHO, CIT, IEG, or LIN at different doses and under different temperature/a_w_ regimes. Incubation time: 21 days.

Output Variable	ML Model Tested	Best Model Parameters	RMSE ^a^	R^2 b^
GR	NN ^c^	size = 5, decay = 0.4	1.444	0.865
	RF ^d^	ntree = 500, mtry = 4	1.260	0.899
	XGBoost ^e^	max_depth = 6, eta = 0.1, subsample = 1	1.160	0.919
	SVM ^f^	C = 1	1.540	0.849
	MLR ^g^		1.708	0.810
T-2 production	NN	size = 2, decay = 0.2	0.569	0.794
	RF	ntree = 500, mtry = 4	0.401	0.903
	XGBoost	max_depth = 2, eta = 0.1, subsample = 1	0.481	0.858
	SVM	C = 1	0.617	0.780
	MLR		0.634	0.772
HT-2 production	NN	size = 2, decay = 0.01	0.589	0.725
	RF	ntree = 500, mtry = 4	0.530	0.781
	XGBoost	max_depth = 2, eta = 0.3, subsample = 1	0.505	0.804
	SVM	C = 1	0.548	0.778
	MLR		0.884	0.762

^a^ RMSE: Root mean squared error for the test set; ^b^ R^2^: R-squared (coefficient of determination) for the test set; ^c^ NN: artificial neural network (single-hidden-layer perceptron); ^d^ RF: random forest; ^e^ XGBoost: extreme gradient boosted tree (the following parameters had constant values: nrounds = 100, gamma = 0, colsample_bytree = 1, min_child_weight = 0.5); ^f^ SVM: support vector machine; ^g^ MLR: multiple linear regression (the best model parameters are in Table S2).

**Table 4:** Main parameters for each ML model applied to the present study and their tuning values.

ML Model	Parameter Name	Description	Tuning Values
Neural network (perceptron) (NN)	size	Hidden layer units (nodes)	[2,5,10,15,20]
	decay	Weight decay rate	[0.01, 0.1, 0.2, 0.3, 0.4]
Random forest (RF)	mtry	Number of randomly selected predictors	[2,3,4]
	ntree	Number of trees	500
Support vector machine (SVM)	C	Cost function	[1,2,3]
Extreme gradient boosted tree (XGBoost)	nrounds	Number of iterations	100
	max_depth	Maximum tree depth	[1,2,3,4,5,6]
	eta	Learning rate (shrinkage)	[0.1, 0.2, 0.3]
	gamma	Regularization penalty factor	0
	colsample_bytree	Fraction of columns to be randomly sampled for each tree	1
	min_child_weight	Minimum instance weight per node	0.5
	subsample	Subsample ratio from training set to grow the trees	[0.5, 0.75, 1]
Multiple linear regression (MLR)	Regression coefficients	Estimates of the constants that multiply the predictor variables	To be determined by software

## Data Availability

The data supporting reported results other than those included in Tables and Figures in the main text or in Supplementary Materials are available, on request, from the corresponding Author.

## Supplementary Materials

The following are available online at https://www.mdpi.com/article/10.3390/toxins13080545/s1, Figure S1: Tuning graph showing the change of RMSE during 10-fold cross-validation of some ML algorithms to model GR of *F. sporotrichioides* on oat grain cultures using EVOH films containing CINHO, CIT, IEG or LIN at different doses (0–3330 µg/culture), under different temperature/a_w_ regimes. Temperatures: 15, 20 and 25 °C; a_w_: 0.96 and 0.99. (a) SVM; (b) NN (one-layer perceptron); (c) RF; (d) XGBoost. The goal was minimizing RMSE, Figure S2. Tuning graph showing the change of RMSE during 10-fold cross-validation of NN, RF and XGBoost algorithms to model production of T-2 and HT-2 toxins in cultures of *F. sporotrichioides* on oat grain using EVOH films CINHO, CIT, IEG or LIN at different doses (0–3330 µg/culture), under different temperature/a_w_ regimes. Temperatures: 15, 20 and 25 °C; a_w_: 0.96 and 0.99. Models for T-2 toxin: (a) NN; (b) RF; (c) XGBoost. Models for HT-2 toxin: (d) NN; (e) RF; (f) XGBoost. Graphics for SVM are omitted because the minimum RMSE was reached for C = 1, as for GR (see Figure S1), Figure S3. Scatter plots and lines of best fit of predicted output values (Y) provided by five predictive ML models versus observed output values (X) for the same test set. The outputs were GR (mm/day) of *F. sporotrichioides* cultures on oat grain incubated with EVOH films containing CINHO, CIT, IEG or LIN at different doses (0–3330 µg/culture) and under different temperature and a_w_ regimes. XGBoost: extreme gradient boosted tree; RF: random forest; SVM: support vector machine; NN: neural network; MLR: multiple linear regression, Figure S4. Scatter plots and lines of best fit of predicted output values (Y) provided by five predictive ML models *versus* observed output values (X) for the same test set. The outputs were T-2 toxin levels produced in *F. sporotrichioides* cultures on oat grain incubated with EVOH films containing CINHO, CIT, IEG or LIN at different doses (0–3330 µg/culture) and under different temperature and a_w_ regimes. XGBoost: extreme gradient boosted tree; RF: random forest; SVM: support vector machine; NN: neural network; MLR: multiple linear regression. Incubation time: 21 days. The decimal logarithms of concentration plus 1 were computed as output values, Figure S5. Scatter plots and lines of best fit of predicted output values (Y) provided by five predictive ML models versus observed output values (X) for the same test set. The outputs were HT-2 toxin levels produced in *F. sporotrichioides* cultures on oat grain incubated with EVOH films containing CINHO, CIT, IEG or LIN at different doses (0–3330 µg/culture) and under different temperature and a_w_ regimes. XGBoost: extreme gradient boosted tree; RF: random forest; SVM: support vector machine; NN: neural network; MLR: multiple linear regression. Incubation time: 21 days. The decimal logarithms of concentration plus 1 were computed as output values, Table S1: Arrangement of GR of *F. sporotrichioides* and accumulation of T-2 and HT-2 toxins in oat kernels in homogeneous groups by post-hoc Duncan’s multiple range test (α = 0.05). The factors are EVOH film type, dose, temperature and a_w_. Incubation time: 21 days. Within each column, the levels containing a X form a homogeneous group of means within which the Duncan’s test does not find statistically significant differences, Table S2. Optimized MS/MS parameters using the UPLC–MS/MS method operating in ESI+ mode for T-2 and HT-2 toxins. Table S3. Values of the regression coefficients obtained using R software for the MLR models developed to predict GR of F. sporotrichioides, and levels of T-2 and HT-2 toxins produced in cultures on oat grain with EVOH films containing CINHO, CIT, IEG and LIN at different doses (0–3330 µg/culture) and under different temperature/aw regimes. Incubation time: 21 days.
