# Supplementary material for: Comparative Analysis of Machine Learning Methods to Predict Growth of F. sporotrichioides and Production of T-2 and HT-2 Toxins in Treatments with Ethylene-Vinyl Alcohol Films Containing Pure Components of Essential Oils
Source: Toxins (Basel). 2021 Aug 5;13(8):545. doi: 10.3390/toxins13080545 (PMC8402422; doi:10.3390/toxins13080545)
Supplement: Supplementary file 1 [file toxins-13-00545-s001.zip › toxins-1321328-supplementary.pdf]

Supplementary Materials: Comparative Analysis of Machine Learning Methods to Predict Growth of *F. sporotrichioides* and Production of T-2 and HT-2 Toxins in Treatments with Ethylene-Vinyl Alcohol Films Containing Pure Components of Essential Oils

Table S1. Arrangement of GR of *F. sporotrichioides* and accumulation of T-2 and HT-2 toxins in oat kernels in homogeneous groups by post-hoc Duncan's multiple range test (□ = 0.05). The factors are EVOH film type, dose, temperature and a_w. Incubation time: 21 days. Within each column, the levels containing a X form a homogeneous group of means within which the Duncan's test does not find statistically significant differences.

Factor	Level	GR					T-2 toxin					HT-2 toxin			
		Low □ high					Low □ high					Low □ high			
EVOH film type	CIT	X					X					X			
	CINHO	X						X						X	
	IEG		X					X					X		
	LIN			X					X				X		
Dose (µg EO component/Petri dish)	333					X					X				X
	666				X					X					X
	1665		X					X				X			
	3330	X					X					X			
Temperature (°C)	28				X		X					X			
	20		X					X				X			
	15	X							X				X		
a_w	0.96	X					X					X			
	0.99	X						X					X		

Table S2. Optimized MS/MS parameters using the UPLC–MS/MS method operating in ESI+ mode for T-2 and HT-2 toxins

									ESI+	
**Mycotoxin**	**Molecular Formula**	**Exact Molecular Mass (Da)**	**Precursor Ion Form**	**Precursor Ion (*m/z*)**	**Product Ion (*m/z*)[a]**	**Cone Voltage (V)**	**Collision Energy (eV)**	**Desolvation Temperature (°C)**	**Source Block Temperature (°C)**	**Capillary Voltage (kV)**
T-2 toxin	$C_{24}H_{34}O_9$	466.527	$[M+NH_4]^+$	484.0	305.0 (Q)	20	15	300	120	3
					245.0 (q)					
HT-2 toxin	$C_{22}H_{32}O_8$	424.210	$[M+NH_4]^+$	442.0	263.0 (Q)					
					215.0 (q)					

[a] Q: quantifier ion; q: qualifier assistant ion

Table S3. Values of the regression coefficients obtained using R software for the MLR models developed to predict GR of *F. sporotrichioides*, and levels of T-2 and HT-2 toxins produced in cultures on oat grain with EVOH films containing CINHO, CIT, IEG and LIN at different doses (0–3330 µg/culture) and under different temperature/a_w regimes. Incubation time: 21 days.

**Output**	**Regression coefficients**		**Significance[a]**
	**Input variable**	**Value**	
GR	Intercept	−4.225	
	Temperature	0.2154	***
	a_w	8.521	
	EVOH film type[b]		
	EVOH-IEG	1.5666	*
	EVOH-LIN	2.6027	***
	EVOH-CIT	0.1613	
	Dose	-2.624×10^{-3}	***
T-2 toxin level	Intercept	−20.50	***
	Temperature	8.425×10^{-3}	
	a_w	23.61	***
	EVOH film type		
	EVOH-IEG	0.3672	*
	EVOH-LIN	0.77	***
	EVOH-CIT	3.326×10^{-2}	
	Dose	-8.09×10^{-4}	***
HT-2 toxin level	Intercept	−21.39	***
	Temperature	-2.766×10^{-2}	*
	a_w	24.53	***
	EVOH film type		
	EVOH-IEG	0.4937	**
	EVOH-LIN	0.8354	***
	EVOH-CIT	6.519×10^{-2}	
	Dose	-6.698×10^{-4}	***

[a] Significance code: ***: *p-value* < 0.001; **: *p-value* < 0.01; *: *p-value* < 0.05 [b] Categorical variable. Recoded in three "dummy" variables (EVOH-IEG, EVOH-LIN and EVOH-CIT).

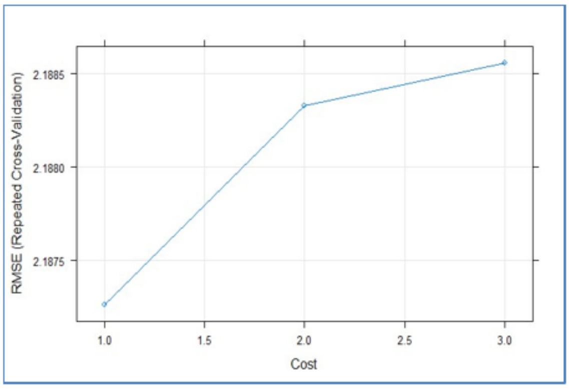

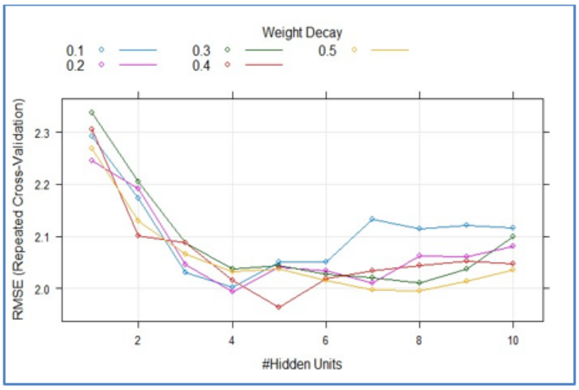

(a) (b)

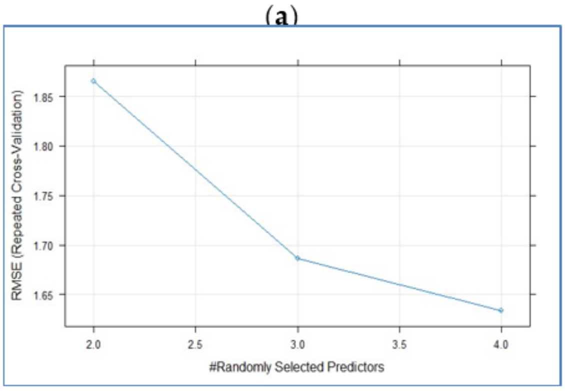

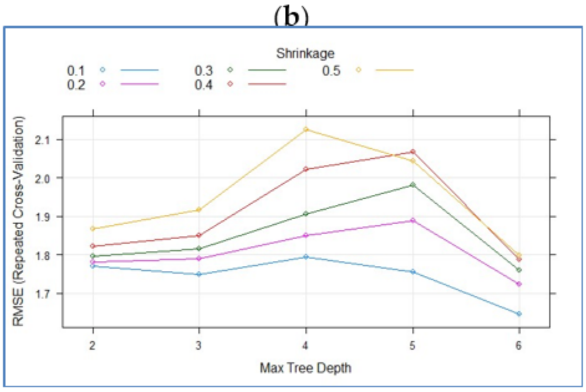

(c) (d)

Figure S1. Tuning graph showing the change of the RMSE during 10-fold cross validation of some ML algoritms to model GR of *F. sporotrichioides* on oat grain cultures using EVOH films containing CINHO, CIT, IEG or LIN at different doses (0 – 3330 μg/culture), under different temperature/a_w regimes.

Temperatures: 15, 20 and 25 °C; a_w: 0.96 and 0.99. (**a**) SVM; (**b**) NN (one-layer perceptron); (**c**) RF; (**d**) XGBoost. The goal was minimizing RMSE.

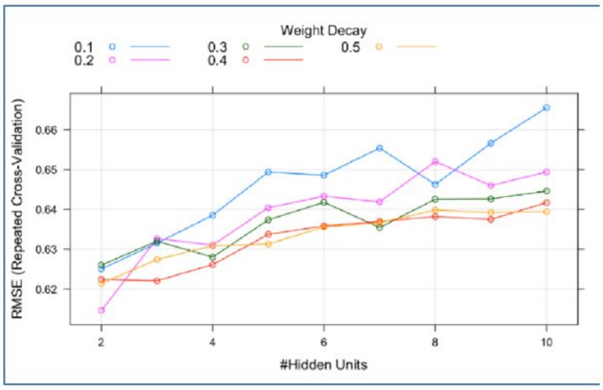

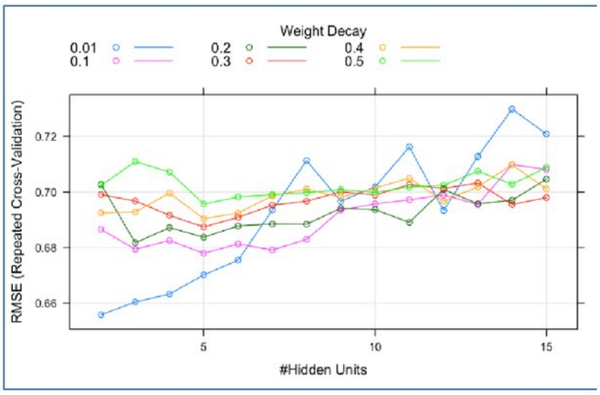

(a) (d)

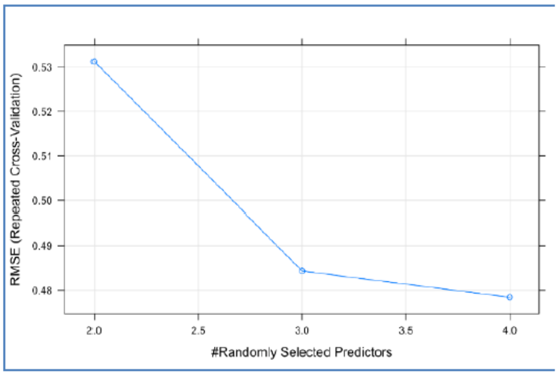

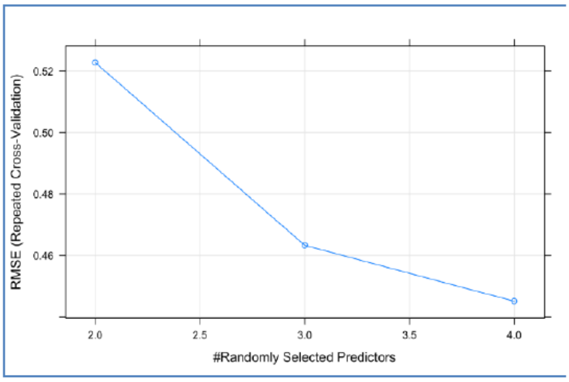

(b) (e)

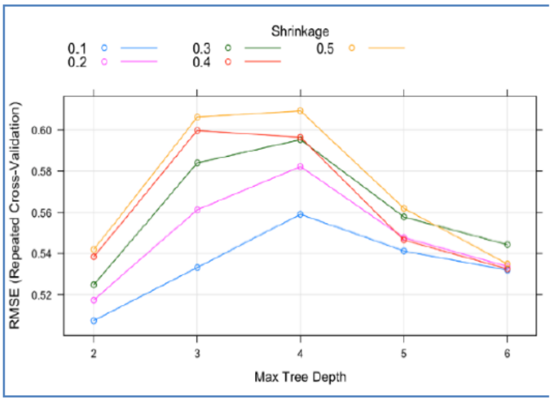

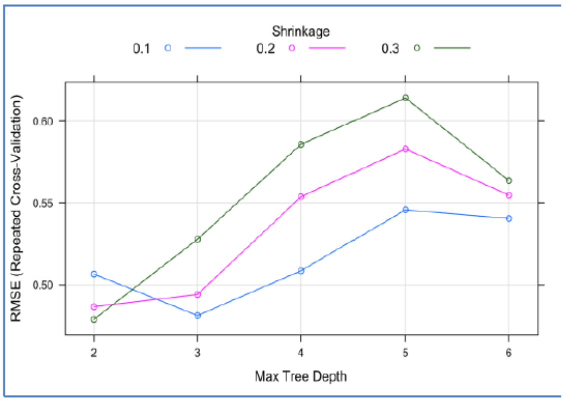

(c) (f)

Figure S2. Tuning graph showing the change of RMSE during 10-fold cross-validation of NN, RF and XGBoost algorithms to model production of T-2 and HT-2 toxins in cultures of *F. sporotrichioides* on oat grain using EVOH films CINHO, CIT, IEG or LIN at different doses (0 – 3330 μg/culture), under different temperature/a_w regimes. Temperatures: 15, 20 and 25 °C; a_w: 0.96 and 0.99. Models for T-2 toxin: (**a**) NN; (**b**) RF; (**c**) XGBoost. Models for HT-2 toxin: (**d**) NN; (**e**) RF; (**f**) XGBoost. Graphics for SVM are omitted because the minimum RMSE was reached for C = 1, as for GR (see Figure S1).

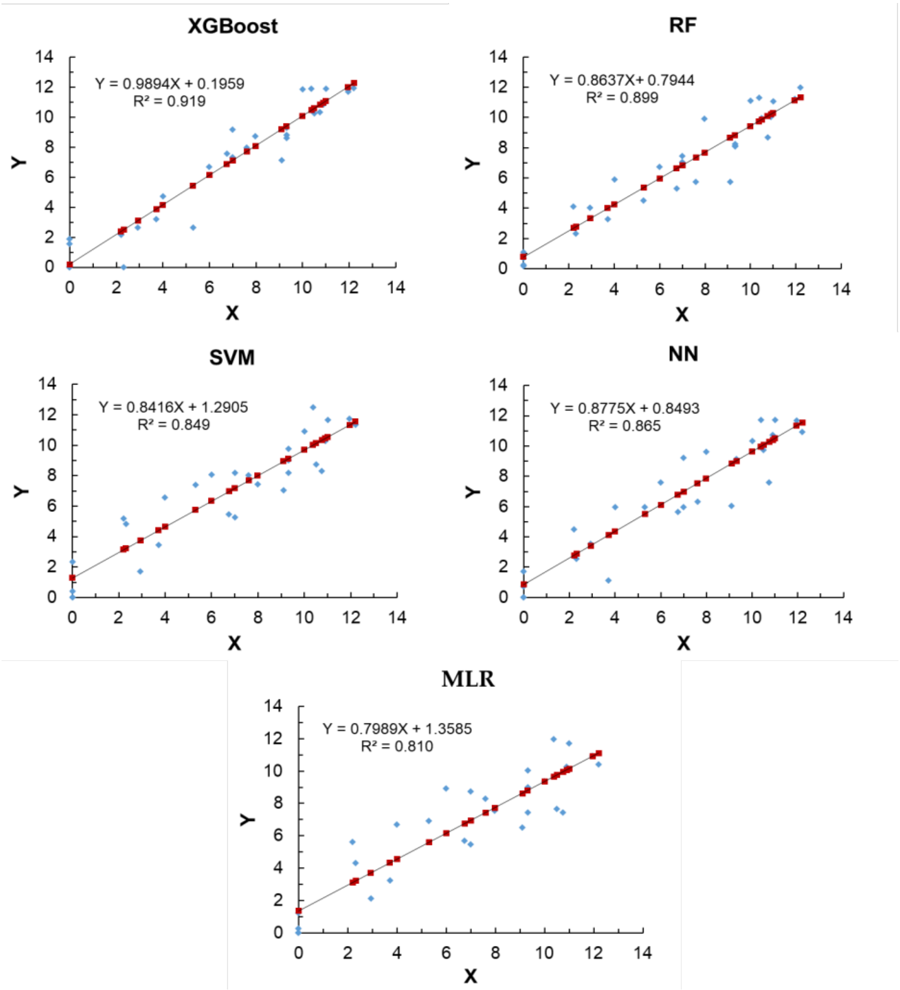

Figure S3. Scatter plots and lines of best fit of predicted output values (Y) provided by five predictive ML models versus observed output values (X) for the same test set. The outputs were GR (mm/day) of *F. sporotrichioides* cultures on oat grain incubated with EVOH films containing CINHO, CIT, IEG or LIN at different doses (0 – 3330 µg/culture) and under different temperature and a_w regimes. XGBoost: extreme gradient boosted tree; RF: random forest; SVM: support vector machine; NN: neural network; MLR: multiple linear regression.

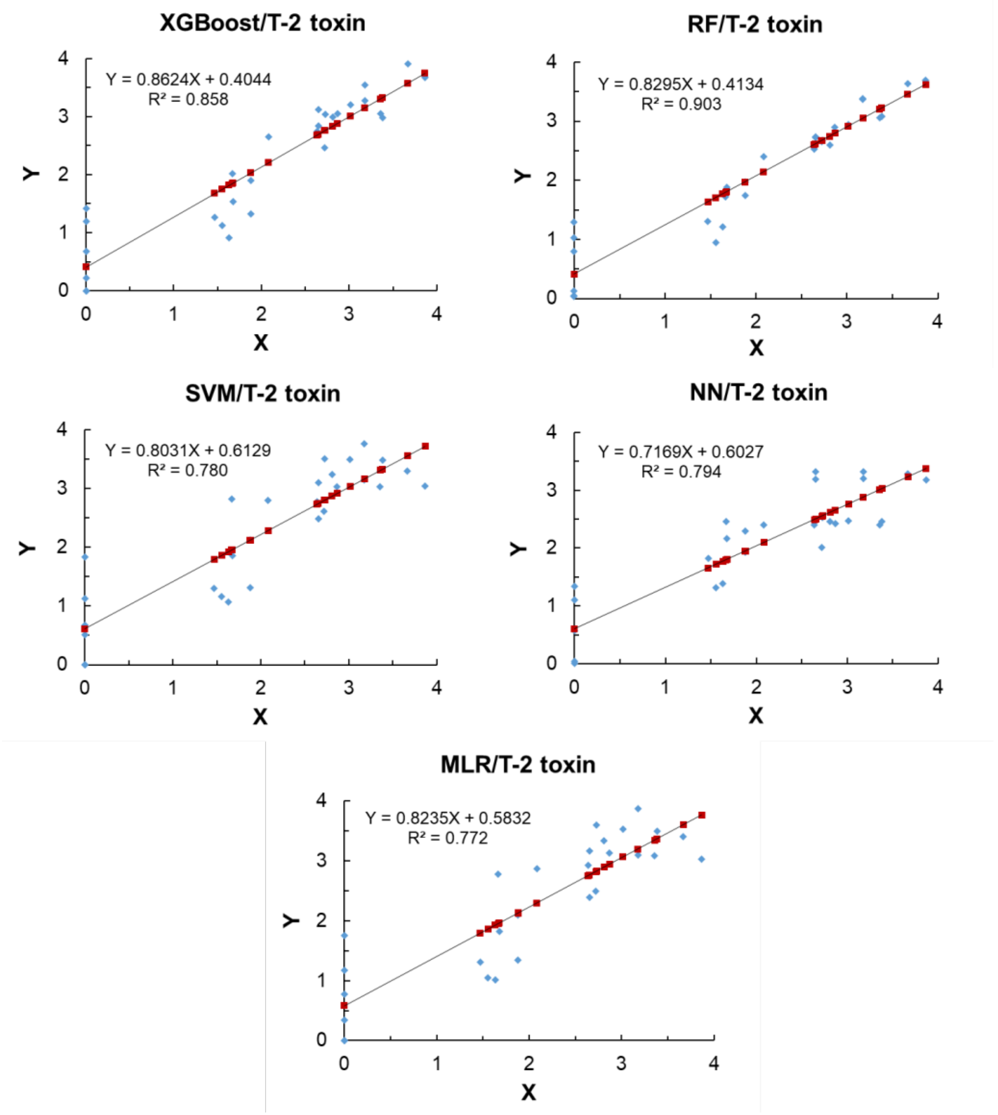

Figure S4. Scatter plots and lines of best fit of predicted output values (Y) provided by five predictive ML models versus observed output values (X) for the same test set. The outputs were T-2 toxin levels produced in *F. sporotrichioides* cultures on oat grain incubated with EVOH films containing CINHO, CIT, IEG or LIN at different doses (0 – 3330 µg/culture) and under different temperature and a_w regimes. XGBoost: extreme gradient boosted tree; RF: random forest; SVM: support vector machine; NN: neural network; MLR: multiple linear regression. Incubation time: 21 days. The decimal logarithms of concentration plus 1 were computed as output values.

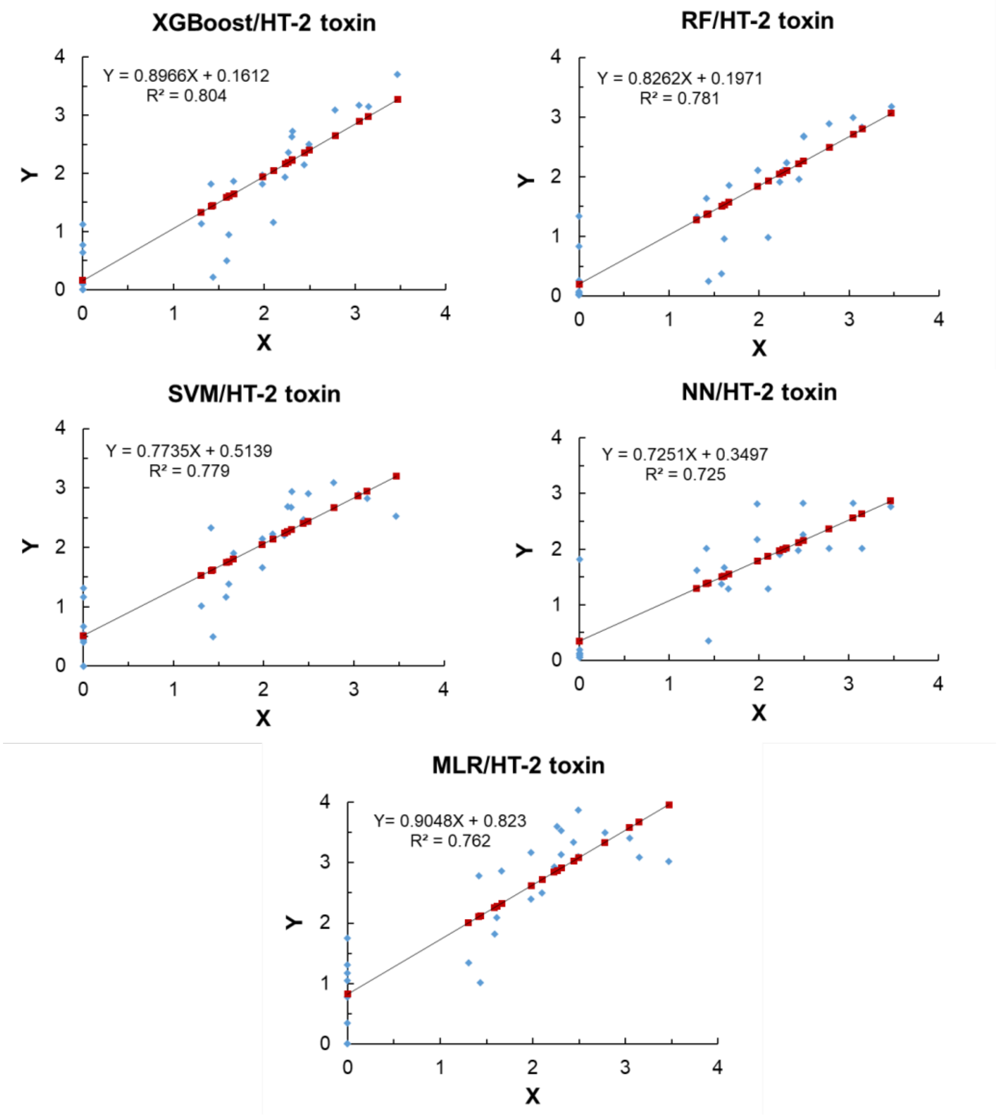

Figure S5. Scatter plots and lines of best fit of predicted output values (Y) provided by five predictive ML models versus observed output values (X) for the same test set. The outputs were HT-2 toxin levels produced in *F. sporotrichioides* cultures on oat grain incubated with EVOH films containing CINHO, CIT, IEG or LIN at different doses (0 – 3330 μg/culture) and under different temperature and a_w regimes. XGBoost: extreme gradient boosted tree; RF: random forest; SVM: support vector machine; NN: neural network; MLR: multiple linear regression. Incubation time: 21 days. The decimal logarithms of concentration plus 1 were computed as output values.
